# Anti-Anaplastic Thyroid Cancer (ATC) Effects and Mechanisms of PLX3397 (Pexidartinib), a Multi-Targeted Tyrosine Kinase Inhibitor (TKI)

**DOI:** 10.3390/cancers15010172

**Published:** 2022-12-28

**Authors:** Jingtao Luo, Yun Wang, Lingkun Zhao, Chunli Wang, Ze Zhang

**Affiliations:** 1 Department of Maxillofacial and Otorhinolaryngology Oncology and Department of Head and Neck Oncology, Tianjin Medical University Cancer Institute and Hospital, National Clinical Research Center for Cancer, Tianjin 300060, China; 2 Tianjin’s Clinical Research Center for Cancer, Tianjin 300060, China; 3 Key Laboratory of Cancer Prevention and Therapy, Tianjin 300060, China

**Keywords:** PLX3397 (Pexidartinib), anaplastic thyroid cancer (ATC), reactive oxygen species (ROS), endoplasmic reticulum (ER) stress, Nrf2

## Abstract

**Simple Summary:**

Anaplastic thyroid cancer (ATC) is the highest lethal type of thyroid cancer. Regrettably, ATC patients respond poorly to multiple treatment strategies. Therefore, there is imperative to strengthen the therapeutic approach to this vicious form of cancer. In the present study, we found that pexidartinib induces ER stress and elevated ROS in ATC cells. The apoptotic cells, and ER stress in ATC after administration of pexidartinib could be reversed by ER stress inhibitor and ROS scavenger, respectively. Furthermore, pexidartinib treatment induced Nrf2 (Nuclear Factor Erythroid 2–related Factor 2) accumulation in nuclei and reduced the interaction of Nrf2 with Keap-1 (Kelch-like ECH-associated protein 1), while the knockdown of Nrf2 enhanced the anti-ATC effects of pexidartinib in vitro. In addition, pexidartinib significantly inhibits ATC xenografts growth and proliferation in vivo, and the combination of ML385, an Nrf2 inhibitor, potently enhanced the anti-ATC effects of pexidartinib in vivo. Our findings suggest pexidartinib to be a potential agent for treating of ATC.

**Abstract:**

Background Anaplastic thyroid cancer (ATC) is the greatest lethal thyroid neoplasm with a low incidence and lacks an effective treatment strategy and standardized treatment protocol. PLX3397 (Pexidartinib) is an FDA-approved multitarget tyrosine kinase inhibitor. The research is designed to explore the possible anti-proliferative activity of pexidartinib on ATC, as well as its related molecular mechanisms. Methods The cell viability was assessed by CCK-8, LDH release, colony formation, and EdU detection assays. Apoptosis and the alteration on cell cycle arrest were characterized by flow cytometry (FCM). ER stress was evaluated by immunofluorescence (IF). ROS levels were determined by flow cytometry. Western blot assays were conducted to evaluate changes in key molecules related to apoptosis and ER stress. The ATC xenografts model was established, and immunohistochemistry was performed to validate the anti-ATC effects of pexidartinib in vivo. Results Pexidartinib significantly inhibited ATC cell proliferation and induced apoptosis and cell cycle arrest. Moreover, pexidartinib potently induced ER stress and elevated ROS in ATC cells, and the apoptotic cells and ER stress in ATC after administration of pexidartinib could be reversed by an ER stress inhibitor and ROS scavenger, respectively. Furthermore, pexidartinib treatment induced Nrf2 accumulation in nuclei and reduced the interaction of Nrf2 with Keap-1, and knockdown of Nrf2 enhanced the anti-ATC effects of pexidartinib in vitro. In addition, pexidartinib significantly inhibited ATC xenograft growth and proliferation in vivo, and the combination of ML385, an Nrf2 inhibitor, potently enhanced the anti-ATC effects of pexidartinib in vivo. Conclusion Our findings suggest pexidartinib is a potential agent for treating ATC. Co-administration with an Nrf2 inhibitor is an effective synergistic strategy.

## 1. Introduction

Though anaplastic thyroid cancer (ATC) is a rare type of thyroid cancer, making up 2% of all thyroid cancers, it is the most lethal. Its median survival time is only 5–12 months because of high rates of extra-thyroidal invasion, distant metastases, as well as resistance to routine therapy [1,2]. Polymodal treatment strategies include surgical resection and hyper-fractionated accelerated external irradiation therapy, in combination with chemotherapy and/or palliative care [3]. Regrettably, ATC patients respond poorly to these treatment strategies [4]. Therefore, it is imperative to strengthen approaches to treat this vicious form of cancer.

PLX3397 (Pexidartinib) is a de novo oral multi-targeted tyrosine kinase inhibitor (TKI). Potential targets of action include the CSF1 receptor, KIT, and FLT3-ITD [5,6,7]. The US Food and Drug Administration (FDA) has authorized pexidartinib capsules as a management strategy for patients with tenosynovial giant cell tumors (TGCTs) who have serious morbidity or functional restrictions that cannot be improved by surgery [8]. In addition, pexidartinib has been proven effective in a series of malignancies. In clinical investigations, pexidartinib monotherapy or, in combination with sirolimus, binimetinib, PLX9486, and durvalumab presented anticancer activity in some solid cancers in adults, including gastrointestinal stromal tumor, pancreatic ductal adenocarcinoma, and colorectal cancer [9,10,11]. In addition, a single administration of pexidartinib was proven effective in antagonizing tumors in children with plexiform neurofibromatosis related to neurofibromatosis type I [12]. These data indicate pexidartinib to be an alternative anticancer agent to other TKIs. However, the anticancer effects of pexidartinib in ATC and its potential mechanisms of action remain to be determined.

The endoplasmic reticulum (ER) conducts and provides an environment for protein folding, as its sensitivity to stimuli can lead to a situation that becomes ER stress, which is considered a well-conserved cellular defense machinery in response to various physiological and pathological events that imbalance ER homeostasis, such as anti-cancer drug-induced apoptosis pathways [13]. The overload status of proteins in the ER is known as ER stress, which can induce unfolded protein response (UPR).

As one of the adverse stimuli, reactive oxygen species (ROS) can contribute to ER dysfunction and lead to endoplasmic reticulum stress, referred to as ROS-mediated ER stress [14]. ROS are important in cellular proliferation and death [15]. In normal metabolic circumstances, appropriate levels of ROS contribute to cell survival. Nevertheless, excessive ROS may cause cell damage and apoptosis [16]. Three molecular signals are involved in UPR, including IRE1, ATF6, and PERK, in which the PERK signal is independent of the other two signals and is involved in ROS-induced apoptosis [17,18,19,20].

In terms of the mechanisms, PERK can phosphorylate eIF2α, which subsequently inhibits the global cellular transcriptional level except for ATF4. Interestingly, the apoptotic cell death can be triggered by ATF4 through CHOP activation, which is a pro-apoptotic molecule associated with cellular tension as well as cell cycle arrest [21]. In anticancer drug discovery, ER stress has been determined to be a ubiquitous course, with a range of TKIs being deployed to initiate the death of apoptotic cells [22]. Therefore, we speculate that pexidartinib should follow a similar mechanism to counteract ATC. In the present study, we show that pexidartinib is effective for treating ATC by activating ER stress via upregulation of ROS levels. The finding provides clues and potential to improve the treatment status of ATC.

## 2. Materials and Methods

### 2.1. Cell Lines, Culturing, and Chemicals

In the present study, human ATC cell lines (CAL-62 and BHT101) were kindly provided by Procell Life Science and Technology (Wuhan, Hubei, China). All cells used in the present study were grown in Dulbecco’s Modified Eagle Medium (DMEM) supplemented with 10% fetal bovine serum (FBS) (Boehringer-Ingelheim, Israel) in a sterile, humidified environment at 37 °C and 5% CO_2_. All cells were test and characterized free of mycoplasm contamination. All cells were identified by STR fingerprinting. Pexidartinib (PLX3397) was purchased from Selleck (Houston, TX, USA).

### 2.2. CCK-8 for Cell Viability Determination

CAL-62 and BHT101 human ATC cells were seeded (5000 cells per well) into 96-well plates in complete DMEM medium and grown overnight. Various concentrations of pexidartinib (ranging from 0–50 μM) were administrated for 24 h to 72 h, as indicated. The CCK-8 assay was conducted according to the manufacturer’s protocol (Beyotime, Shanghai, China). The absorbance value (OD) was measured at 450 nm by an iMark multiplate reader (Bio-Rad, CA, USA). Relevant survival rate of the cells was derived from the following formula: Cell viability = (OD_pexidartinib group_ − OD_blank_)/(OD_control group_ − OD_blank_) × 100. The median inhibitory concentrations (IC_50_) were calculated by GraphPad software (San Diego, CA, USA).

### 2.3. LDH Cell Cytotoxicity Assay

CAL-62 and BHT101 human ATC cells were harvested and seeded into 96-well plates with complete DMEM medium and grown overnight (5000 cells per well). Various concentrations of pexidartinib (ranging from 0–50 μM) were administrated for 24 h, 48 h, and 72 h. The LDH Cytotoxicity Assay kit (Beyotime, Shanghai, China), which determines cytotoxicity based on a colorimetric indicator of lactate dehydrogenase activity released into the cell culture medium, was conducted according to the manufacturer’s instructions. The cellular OD values were detected at 490 nm by an iMark multiplate reader (Bio-Rad, Hercules, CA, USA).

### 2.4. Plate Cloning Assay

CAL-62 and BHT101 human ATC cell lines were harvested and seeded into six-well plates (400 cells per well) for culturing overnight. Cells were then treated with different concentrations of pexidartinib in complete DMEM medium. After another 24 h incubation, the medium was replaced with freshly prepared DMEM medium (10% FBS) for another 10 days of incubation time. Methanol was used to fix the ATC cells at day 10. After cells were washed twice with PBS, crystal violet buffer (0.05%) was used to stain the cells and the formative colonies were identified with cells more than fifty in a colony. Colonies were then counted and the survival colony rates were calculated as previously described [23].

### 2.5. EdU (5-Ethynyl-2′-deoxyuridine) Assay

CAL-62 and BHT101 cell lines were harvested and plated into six-well plates (5 × 10^4^ cells per well) for culturing overnight, then cocultured with various doses of pexidartinib for 24 h. EdU detection to measure cell proliferation was conducted following the manufacturer’s method (Beyotime, Shanghai, China). In brief, the cells were washed, then fixed in 4% paraformaldehyde for 0.5 h. Subsequently, cells were permeated with 0.3% TritonX-100 in PBS, then incubated with the reaction solution. The results were captured by a DMi1 inverted fluorescence microscope (Leica, Wetzlar, Germany).

### 2.6. Apoptotic Cell Detection

The apoptotic cells in CAL-62 and BHT101 human ATC cells were determined by flow cytometry. CAL-62 and BHT101 human ATC cells were harvested (in trypsin without EDTA) and centrifuged after incubating with pexidartinib for 24 h, then re-suspended in binding buffer according to the Annexin V-FITC kit (Beyotime, Shanghai, China). Annexin V-FITC (5 μL) and PI (10 μL) were mixed into binding buffer for 20 min of incubation time. The cells were collected and analyzed by FACSCanto (Becton Dickinson, Franklin Lakes, NJ, USA). The apoptotic cells were identified by FlowJo software 10.8 (TreeStar, Ashland, CA, USA).

### 2.7. Evaluating Caspase-3 Activity

The Caspase 3 Assay Kit (Sigma-Aldrich, St. Louis, MO, USA) was conducted to determine the alteration of caspase-3 activity of CAL-62 and BHT101 cells after pexidartinib treatment for 24 h. In brief, CAL-62 and BHT101 cell lines were plated into six-well plates and grown overnight. Investigation of caspase-3 activity followed the kit-accompanied instructions. Fluorescence values were detected at 405 nm by a VF microplate reader (Thermo Fisher Scientific, Waltham, MA, USA).

### 2.8. Cell Cycle Evaluation

We used flow cytometry to evaluate the impact of pexidartinib on cell cycle alteration in ATC. CAL-62 or BHT101 cells were harvested and then plated into six-well culture plates and incubated with various concentrations of pexidartinib for 24 h. The cells were then collected, centrifuged, and suspended in PBS, followed by fixing in 75% iced alcohol at 4 °C for overnight. Cells were then washed in PBS and resuspended in binding buffer containing 50 μg/mL PI and 100 μg/mL RNAase (Solabio, Beijing, China). The results were obtained by FACSCanto (Becton Dickinson, NJ, USA), and the cell cycle distribution was illustrated by Modifit software LT5.0 (Solvusoft Corporation, Las Vegas, NV, USA).

### 2.9. ROS Determination

A ROS Analysis Kit (Beyotime, Shanghai, China) was utilized according to the manufacturer’s protocol to evaluate the ROS level of CAL-62 and BHT101 cells after pexidartinib treatment. In brief, after various periods of pexidartinib administration, CAL-62 and BHT101 cells were incubated in complete DMEM medium containing 0.1% DCFH-DA for 30 min. Subsequently, cells were either harvested, collected via a FACSCanto (Becton Dickinson, NJ, USA), or imaged using a DMi1 inverted fluorescence microscope (Leica, Wetzlar, Germany).

### 2.10. Immunofluorescence (IF)

CAL-62 and BHT101 cells were seeded in six-well plates on sterile glass cover slips and treated with pexidartinib for 24 h. Then, cells were fixed with 4% paraformaldehyde and permeabilized. Subsequently, cells were washed, and the heterogenetic antigens were blocked by BSA (5%), before incubation with Alexa Fluor^®^ 594 Conjugate-PDI (C81H6) antibody (1:50) at 4 °C for 8 h. Cell images were captured using the FV3000 confocal microscope (Olympus, Tokyo, Japan).

### 2.11. Cell Transfection

Knockdown of Nrf2 was conducted by transfecting small interfering Nrf2 sequences into CAL-62 and BHT101 cells. The siNrf2 sequences were previously described [24]. The sense and antisense sequences are 5′-GGAGGCAGAUAUGUCUTT-3′ and 5′-AGAUCUAUAUCUUGCCUCCTT-3′, respectively. In brief, CAL-62 and BHT101 cells in the logarithmic growth phase were plated into six-well plates. When the cells grew to about 60–70% confluence, the cells were incubated with 2.5 μg of siNrf2 or 2.5 μg of siControl with 5 μL of Lipofectamine^TM^ 3000 Reagent (Invitrogen, Carlsbad, CA, USA). Western blot was conducted to validate the efficiency of Nrf2 knockdown after 48 h transfection. The transfected cells were used for further experiments.

### 2.12. Western Blot Assay

Western blot was used to determine the alteration of pexidartinib on apoptosis as well as ER stress molecular pathways, as previously described [25]. Briefly, after incubation with pexidartinib for 24 h, cells were collected, centrifuged, and resuspended in RIPA lysis buffer containing PMSF as well as phosphatase inhibitor and incubated on ice for 30 min. The components were then centrifuged at 14000× *g* for 15 min to collect the supernatant. A BCA assay was conducted to determine the quantity of the protein. After the protein quantity of the samples was normalized, proteins (20 μg per sample) were subjected to SDS-PAGE and electronically transferred onto PVDF membranes (Merck Millipore, Billerica, MA, USA). BSA (5%) was used to block the non-specific binding. Primary antibodies were incubated overnight at 4 °C. Membranes were washed with TBST buffer, then incubated with appropriate secondary antibodies for 2 h at RT. The protein bands were visualized by enhanced chemiluminescence (ECL) (ThermoFisher Scientific, Waltham, MA, USA). The signals were captured by photographic film in a dark room.

### 2.13. Xenografts and Immunohistochemistry

The protocol of the animal experiment was authorized by the Experimental Ethics Committee of Tianjin Medical University Cancer Institute and Hospital (approval No: PMIS-2020-031). BALB/Ca nude male mice (5 weeks old) were obtained from National Institutes for Food and Drug Control (Beijing, China). All experimental mice had free available accessibility to food and water and were maintained in specific pathogen-free environments at 24 ± 2 °C with a 12 h light/dark cycle. CAL-62 cells (1 × 10^7^ cells in 100 μL) were subcutaneously injected into the right limbs. When tumor volumes reached 50–100 mm^3^, the mice were then randomly divided into four groups, the control group (intragastric and intraperitoneal administration of vehicle), the ML385 group (30 mg/kg intraperitoneally administered, daily), the pexidartinib group (40 mg/kg intragastric administration, daily), and the ML385 and pexidartinib combination group (daily administrated). The concentrations of these molecules were determined as detailed in previous studies [26,27]. The tumor volumes were measured every three days and the treatment lasted for 21 days.

The excised xenografts were fixed in 4% paraformaldehyde. Samples were then dehydrated by increasing concentrations of xylene, prior to being embedded in paraffin, then sectioned (4 μm). After incubation in hydrogen peroxide (3%), the antigen retrieval was conducted by incubating the samples in EDTA antigen retrieval buffer (ZLI-9066, ZSGB-BIO, Beijing, China) through continuous boiling in a microwave for 10 min. The samples were then incubated with 10% goat serum and were subsequently incubated with primary antibody (Ki-67, 1:100, TA800648, ZSGB-BIO, Beijing, China). The samples were washed and incubated with secondary antibody following marked by DAB (ZSGB-BIO, Beijing, China). A Leica LED Binocular Microscope (Leica, Wetzlar, Germany) was used to capture the results. The IHC scores were evaluated as previously described using ImageJ software to define the Ki67 positive cells [28].

### 2.14. Statistical Analysis

The data shown in bar plots were generated using GraphPad 9.00 and are expressed as mean ± SD in at least three independent duplicates. Student’s t-test analysis was applied to determine the statistical significance when comparing two groups. Comparisons of multigroups were determined by ordinary One-Way Analysis of Variance (ANOVA) with Student-Newman–Keuls test when the comparing three groups. For groups more than three, Tukey’s test was conducted. Statistical significance was considered as *p*-value < 0.05 (shown as *).

## 3. Results

### 3.1. Pexidartinib Antagonizes ATC Cell Proliferation

**Figure 1:**
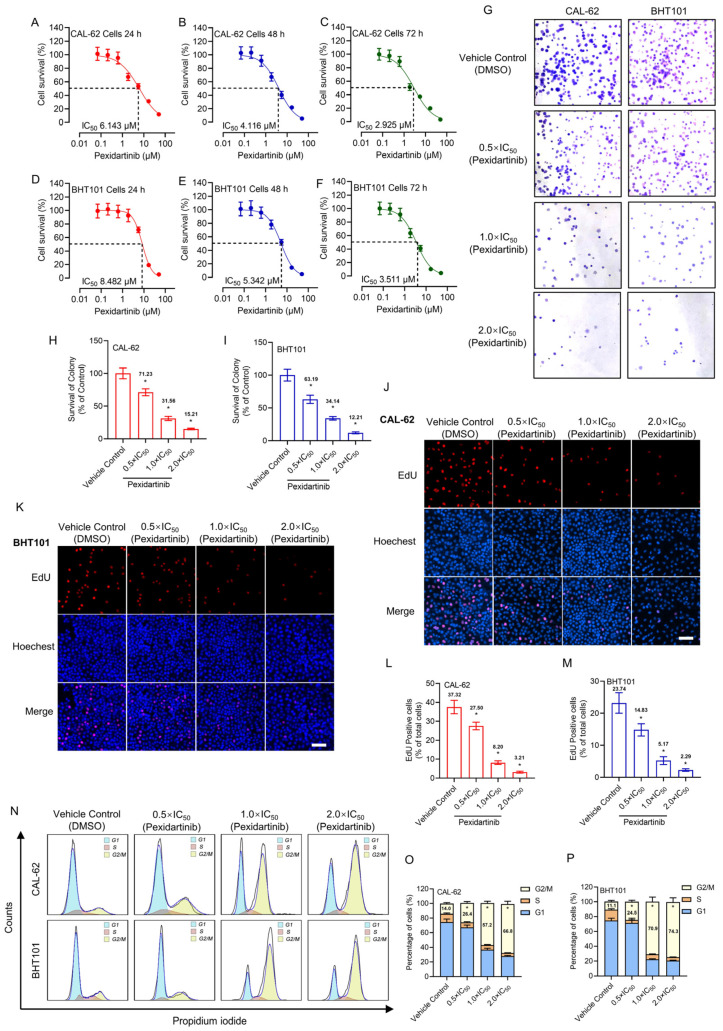
Pexidartinib inhibits ATC cells proliferation: (**A**–**C**) Viability-survival curves of CAL-62 cells treated with different concentrations of pexidartinib for 24 h, 48 h, and 72 h, respectively. (**D**–**F**) Viability-survival curves of BHT101 cells treated with pexidartinib for 24 h, 48 h, and 72 h, respectively. (**G**) Colony formation assays of CAL-62 and BHT101 cells treated with pexidartinib at 0.5 × IC_50_, 1 × IC_50_, and 2×IC_50_ concentrations. (**H**,**I**) Relative survival of colony detected in CAL-62 and BHT101 cells treated with pexidartinib at 0.5 × IC_50_, 1 × IC_50_, and 2 × IC_50_ concentrations. (**J**–**M**) EdU detection in CAL-62 and BHT101 cells treated with pexidartinib at 0.5 × IC_50_, 1 × IC_50_, and 2 × IC_50_ concentrations. (**N**) Cell cycle distribution after pexidartinib (0.5 × IC_50_, 1 × IC_50_, and 2 × IC_50_ concentrations) treatment in CAL-62 and BHT101 cells. (**O**,**P**) Statistical analysis of cell cycle distribution after pexidartinib (0.5 × IC_50_, 1 × IC_50_, and 2 × IC_50_ concentrations) treatment in CAL-62 and BHT101 cells. Data are presented as mean ± SD in at least three independent experiments (significance is presented as * when *p* < 0.05).

The anti-tumor characteristics of pexidartinib on CAL-62 and BHT101 cells were evaluated by CCK-8, plate colony formation, and EdU assays. As illustrated in Figure 1A–C, pexidartinib showed potent anti-proliferative effects on CAL-62 cells in dose- and time-dependent manners. The IC_50_ values of pexidartinib on CAL-62 cells were 6.413 μM (24 h incubation), 4.116 μM (48 h incubation), and 2.925 μM (72 h incubation). For the BHT101 cells, pexidartinib also showed potent anticancer effects. For the 24 h incubation, the IC_50_ values of pexidartinib on BHT101 cells were 8.482 μM, for the 48 h and 72 h treatments, and the IC_50_ values of pexidartinib were 5.342 μM and 3.511 μM, respectively (Figure 1D–F).

The alteration of clonality of pexidartinib on CAL-62 and BHT101 cells was also evaluated. We chose three concentrations of pexidartinib according to the IC_50_ values determined for both cell lines, which were half times IC_50_ value (0.5 × IC_50_), IC_50_ value (1 × IC_50_), and two times IC_50_ value (2 × IC_50_), respectively. As illustrated in Figure 1G, the colony numbers in both CAL-62 and BHT101 cells were potently decreased after pexidartinib incubation. Statistical analysis indicated that pexidartinib inhibited the colony formation of both CAL-62 and BHT101 cells in a dose-dependent manner (Figure 1H,I). Similar results were also observed in the EdU assays. As shown in Figure 1J–M, after administration of increasing concentrations of pexidartinib, the EdU-positive CAL-62 and BHT101 cells potently decreased. Statistical analysis indicated that pexidartinib significantly suppress the proliferation-related characteristics of CAL-62 and BHT101 cells concentration dependently. In addition, the results illustrated in Figure 1N–P indicated that pexidartinib also induced G2/M phase cell cycle arrest in CAL-62 and BHT101 cells. These results fully confirm that pexidartinib significantly inhibits the proliferative activity in ATC cells and exhibits dose-dependent and time-dependent characteristics.

### 3.2. Pexidartinib Illustrates Cytotoxicity Effects and Increases Apoptotic Cells in ATC

**Figure 2:**
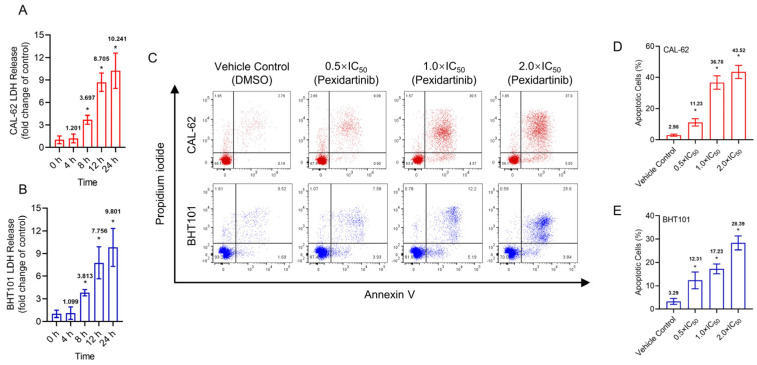

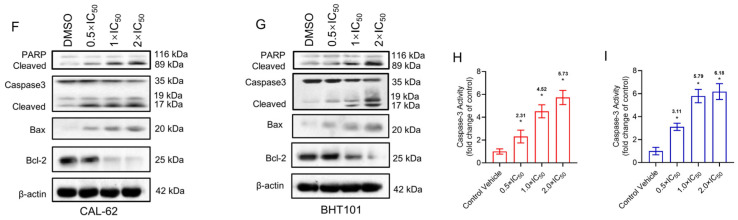
Pexidartinib induces cytotoxicity and apoptosis in ATC cells: (**A**,**B**) LDH release detected in CAL-62 and BHT101 cells after administration of pexidartinib at IC_50_ concentrations for various times. (**C**) Dot blots show the apoptotic cells after pexidartinib treatment in CAL-62 and BHT101 cells respectively at 0.5 × IC_50_, 1 × IC_50_, and 2 × IC_50_ concentrations. (**D**,**E**) The statistical analysis of pexidartinib-induced apoptosis in CAL-62 and BHT101 cells, respectively. (**F**,**G**) Western blot assays show the alteration of apoptosis-related proteins after pexidartinib (0.5 × IC_50_, 1 × IC_50_, and 2 × IC_50_ concentrations) treatment in CAL-62 and BHT101 cells, respectively. (**H**,**I**) Caspase-3 activity detected in CAL-62 and BHT101 cells after pexidartinib (0.5 × IC_50_, 1 × IC_50_, and 2 × IC_50_ concentrations) treatment. The original blots can be found in Figure S1. Data are presented as mean ± SD in at least three independent experiments (significance is presented as * when *p* < 0.05).

Pexidartinib potently induced the LDH release in both CAL-62 and BHT101 cells, the earliest significant change of LDH in both cells was at 8 h (Figure 2C–E). To identify the possible mechanisms of pexidartinib-induced ATC cell death, apoptotic cells were detected after pexidartinib administration. After incubation with increasing concentrations of pexidartinib, the cell dots in the second and third quadrant obviously increased, and the statistical analysis indicated that pexidartinib significantly induced apoptosis in CAL-62 and BHT101 cells dependent upon concentration (Figure 2C–E). The apoptotic-related proteins were also evaluated. As shown in Figure 2F,G, cleaved PARP and caspase-3 increased after administration with various doses of pexidartinib in both CAL-62 and BHT101 cells. In addition, the expression level of Bax, a representative pro-apoptotic molecule, significantly increased, while Bcl-2, an anti-apoptotic protein, potently decreased under pexidartinib treatment. As shown in Figure 2H,I, pexidartinib significantly upregulated the activity of caspase-3 in both CAL-62 and BHT101 cells dose-dependently.

### 3.3. ER Stress Is Responsible for the Apoptosis Induced by Pexidartinib

**Figure 3:**
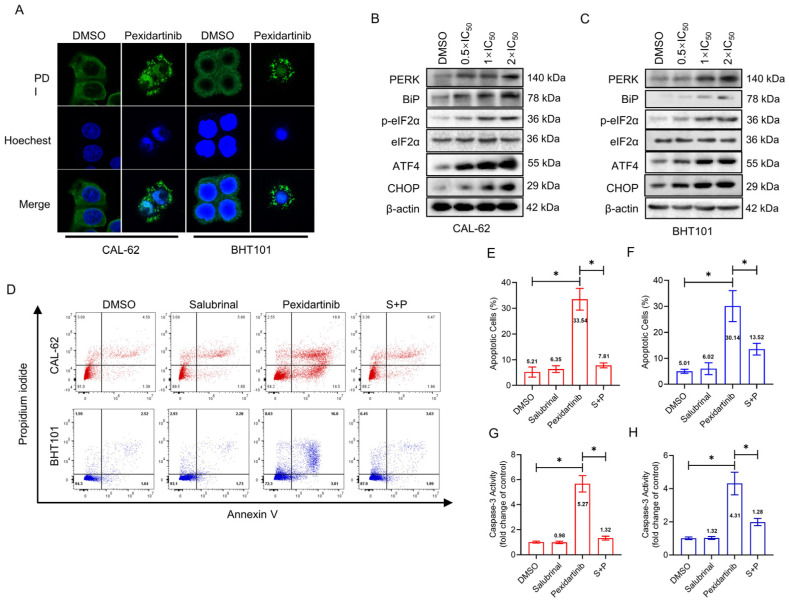
Pexidartinib induces apoptosis in ATC cells in an ER stress-dependent manner: (**A**) Immunofluorescence detection of protein disulphide isomerase (PDI). (**B**,**C**) PERK-dependent molecular pathway protein alteration in ER stress detected by Western blot. (**D**) Apoptotic cells detection in CAL-62 and BHT101 cells after salubrinal treatment or combination of salubrinal with pexidartinib. (**E**,**F**) Statistical analysis of apoptotic cells detected in CAL-62 and BHT101 cells after salubrinal or combination of salubrinal with pexidartinib. (**G**,**H**) Alteration of Caspase-3 activity determined in CAL-62 and BHT101 cells after salubrinal or combination of salubrinal with pexidartinib. The original blots can be found in Figure S1. Data are presented as mean ± SD in at least three independent experiments (significance is presented as * when *p* < 0.05).

The alteration of specific markers in the ER cavity protein disulphide isomerase (PDI) was evaluated by IF after administration of pexidartinib. As illustrated in Figure 3A, the obvious aggregation of PDI was observed in both the CAL-62 and BHT101 cells after 24 h administration with pexidartinib, indicating that pexidartinib might initiate ER stress in ATC cells. Moreover, we evaluated the expression of protein involved in ER stress signaling, and as shown in Figure 3B,C, the key molecules in ER stress signaling pathways were potently increased with increasing dose administration of pexidartinib, including PERK, Bip, phosphorylated-eIF2α, ATF4, and CHOP. In addition, salubrinal, which is an inhibitor of eIF2α dephosphorylation, significantly reversed the apoptosis induced by pexidartinib (Figure 3D–F). Moreover, the up-regulation of caspase-3 in ATC cells after pexidartinib incubation could be also reversed by salubrinal treatment, indicating that inhibiting ER stress could reverse salubrinal-induced apoptosis in ATC cells (Figure 3G,H). These results suggested that ER stress could be a core mechanism of pexidartinib-induced ATC cell apoptosis.

### 3.4. Pexidartinib Induces ER Stress and Accompanying Apoptosis in ATC Cells by Elevation of Intracellular ROS

**Figure 4:**
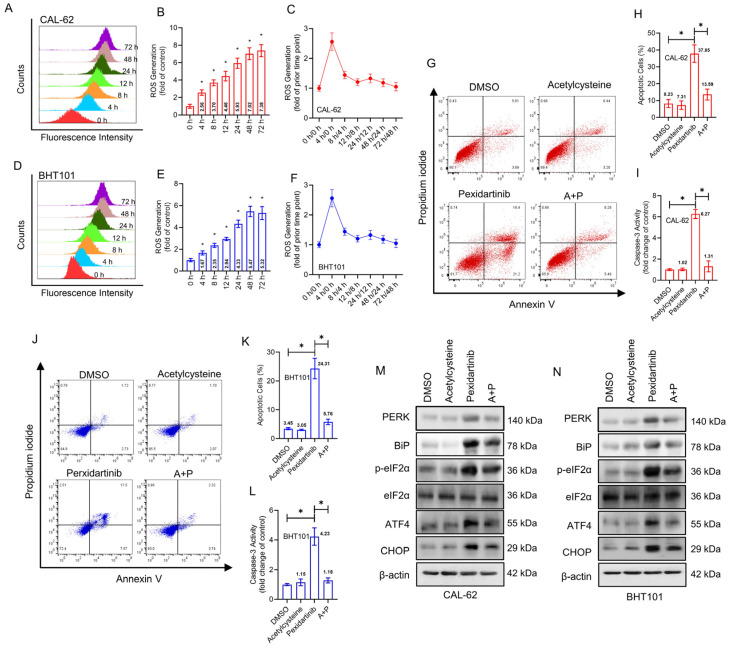
Pexidartinib induces ROS elevation in ATC cells. (**A**,**B**) ROS levels determined after treatment with pexidartinib at IC_50_ concentration for various time points in CAL-62 cells. (**C**) Change in ROS generation was determined after treatment with pexidartinib at IC_50_ concentration for various time points in CAL-62 cells. (**D**,**E**) ROS level was determined after treatment with pexidartinib at IC_50_ concentration for various time points in BHT101 cells. (**F**) Change in ROS generation was determined after treatment with pexidartinib at IC_50_ concentration for various time points in BHT101 cells. (**G**–**I**) Apoptotic cells detected in CAL-62 and cells after acetylcysteine or combination of acetylcysteine with pexidartinib. (**J**–**L**) Apoptotic cells were detected in CAL-62 and cells after acetylcysteine or combination of acetylcysteine with pexidartinib. (**M**,**N**) PERK-dependent molecular pathways protein alteration on ER stress detected by Western blot after acetylcysteine or combination of acetylcysteine with pexidartinib. The original blots can be found in Figure S1. Data are presented as mean ± SD in at least three independent experiments (significance is presented as * when *p* < 0.05).

To deeply investigate the mechanisms of pexidartinib-induced ER stress and apoptosis, we determined the intracellular ROS level by flow cytometry using DCFH-DA labeling. As illustrated in Figure 4A,B, the fluorescence intensity of DCFH-DA treated CAL-62 cells increased in different time periods of pexidartinib incubation, showing a time-dependent effect. In addition, ROS grew fastest in the first four hours of pexidartinib incubation, after which the rate of growth stabilized (Figure 4C). Similar results were also observed in another ATC cell line, BHT101, in which ROS increased time-dependently, and the rate tended to be stable after the first 4 h of rapid production (Figure 4D–F). As shown in Figure 4G,H, when the ROS scavenger acetylcysteine was administered with pexidartinib and the apoptotic cells and the activity of caspase-3 were determined, the combination of pexidartinib and acetylcysteine significantly decreased the apoptotic cells in CAL-62 cell line compared to single use of pexidartinib. Moreover, co-administration of pexidartinib and acetylcysteine potently inhibited the caspase-3 activity (Figure 4I). Similar results were also observed in BHT101 cells (Figure 4J–L). Next, we determined whether pexidartinib-induced ER stress could be reversed by acetylcysteine. The expression level of key molecules in ER stress pathways were significantly decreased after co-administration of pexidartinib and acetylcysteine compared to pexidartinib alone (Figure 4M,N). These results suggested that ROS is a crucial factor in pexidartinib-induced ER stress and apoptosis in ATC cells.

### 3.5. Knockdown of Nrf2 Elevates Anticancer Activity of Pexidartinib in ATC Cells

**Figure 5:**
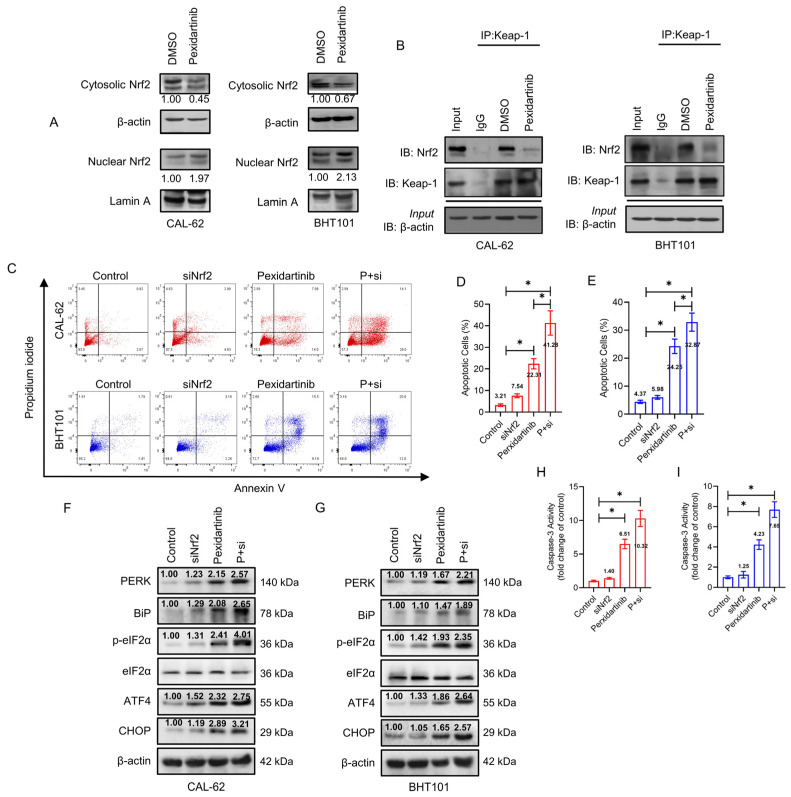
Knockdown of Nrf2 enhances the anti-ATC effects of pexidartinib. (**A**) Western blot analysis for the detection of cytosolic and nuclear levels of Nrf2 expression after pexidartinib administration in CAL-62 and BHT101 cells. (**B**) Co-immunoprecipitation (co-IP) determination to detect interaction of Nrf2 with Keap-1 after pexidartinib administration in CAL-62 and BHT101 cells. (**C**–**E**) Apoptotic cells were detected in CAL-62 and BHT101 cells after knockdown of Nrf2. (**F**,**G**) PERK-dependent molecular pathways protein alteration on ER stress detected by Western blot after administration of pexidartinib in Nrf2-knockdown CAL-62 and BHT101 cells. (**H**,**I**) Caspase-3 activity detected after administration of pexidartinib in Nrf2-knockdown CAL-62 and BHT101 cells. The original blots can be found in Figure S1. Data are presented as mean ± SD in at least three independent experiments (significance is presented as * when *p* < 0.05).

Nrf2 is a representative means of defense on oxidative stress, which could be degraded by ubiquitination in normal environment. We hence speculated that Nrf2 may play key role in pexidartinib-induced ROS increase and ER stress initiation. As illustrated in Figure 5A, pexidartinib potently suppressed the expression level of cytosolic Nrf2, but upregulated nuclear Nrf2 in both CAL-62 and BHT101 cells. Furthermore, the interaction of Nrf2 and Keap-1 was broken after pexidartinib treatment in ATC cells (Figure 5B). We then used siRNA to knockdown Nrf2 in ATC cells and found that the knockdown of Nrf2 extremely enhanced the number of apoptotic cells in both CAL-62 and BHT101 cell lines. In addition, the caspase-3 activity was also enhanced after pexidartinib treatment in both cell lines (Figure 5C–E). Finally, the results of Western blot indicated that knockdown of Nrf2 in both CAL-62 and BHT101 cells potently enhanced pexidartinib-induced ER stress (Figure 5F–I). These results suggested that Nrf2 is a protective mechanism for ATC cells under pexidartinib administration.

### 3.6. Pexidartinib Inhibits ATC Tumor Growth In Vivo, Co-Administration with Nrf2 Inhibitor Enhance the Anti-ATC Effects of Pexidartinib in Vivo

**Figure 6:**
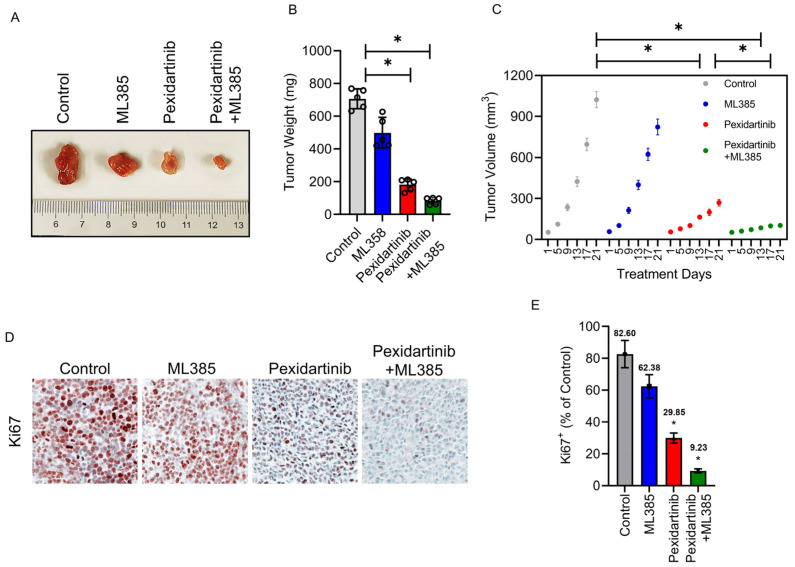
Pexidartinib inhibits ATC proliferation in vivo. (**A**) Images of CAL-62 xenografts after administration of pexidartinib or co-administration of pexidartinib with ML385 (*n* = 5). (**B**) The tumor weights at the end of the treatment (*n* = 5). (**C**) Tumor volume-time curves for xenografts for the treatment period (*n* = 5). (**D**) IHC of Ki67 staining in xenografts. (**E**) Statistical analysis of Ki67 positive cells in xenografts. Data are presented as mean ± SD in at least three independent experiments (significance is presented as * when *p* < 0.05).

To further verify the anticancer characteristics of pexidartinib on ATC in vivo, we established an ATC cell xenograft model. As illustrated in Figure 6A–C, treatment with pexidartinib (40 mg/kg) significantly inhibited the tumor growth and decreased the tumor weight in BALB/Ca nude mice. Co-administration of pexidartinib (40 mg/kg) with the Nrf2 inhibitor ML385 (30 mg/kg) potently inhibited tumor growth compared to the single use of pexidartinib, which agreed with our in vitro results. To evaluate the proliferation activity in the graft, we determined Ki67 expression by IHC. As illustrated in Figure 6D,E, after administration of pexidartinib (40 mg/kg), the percentage of Ki67 positive cells significantly decreased in CAL-62 xenografts, and co-administration of pexidartinib and ML385 further reduced the percentage of Ki67 positive cells. These results suggested that pexidartinib inhibits ATC in vivo, which is synergistically enhanced by the Nrf2 inhibitor.

## 4. Discussion

Over the past few decades, thyroid cancer has become an increasingly significant public health issue in most regions of the world [29]. Among thyroid cancers, ATC is a highly lethal cancer, for which treatment strategies have not yet been universally acknowledged, nor is there identified any highly effective treatment option [30]. Hence, the determination of effective treatment agents targeting ATC as well as the discovery of relevant original therapeutic mechanisms are essential to improving the prognosis of ATC patients. Pexidartinib is an orally administered, small-molecule, multi-kinase inhibitor that selectively inhibits CSF1 receptor, KIT, and FLT3-ITD activity [7]. The FDA has approved pexidatinib capsules for the management of patients with symptomatic tenosynovial giant cell tumors (TGCT) who have critical prevalence or functionality restrictions that cannot be improved by surgery [8]. Recently, it has been documented that pexidartinib inhibits follicular thyroid cancer (FTC) tumor cell proliferation by suppressing the expression of critical cytokine and inflammation-regulating genes, indicating the treatment potential of pexidartinib for thyroid cancer [27]. However, there are no reports of the anti-proliferative effects of pexidartinib on ATC or the related mechanisms.

In the present study, our main finding is that pexidartinib can directly inhibit ATC cells through ROS-mediated ER stress. We first evaluated the inhibition of proliferation by pexidartinib on ATC cells using a variety of assays, including CCK-8, LDH release, colony formation, and EdU detection. Pexidartinib showed potent concentration- and time-dependent inhibition on ATC cell proliferation. To explore further the mechanisms of action on pexidartinib-induced ATC cell death, flow cytometry assays for apoptotic cells detection and cell cycle arrest evaluation were conducted. The results indicated that pexidartinib significantly elevated apoptotic cells and induced cell cycle arrest in ATC cell lines. Mechanistically, the anti-apoptotic molecule Bcl-2 was reduced by pexidartinib, and the upregulation of BAX, a pro-apoptotic protein, was observed after administration of pexidartinib. In addition, cleaved caspase-3 and cleaved PARP were also elevated by pexidartinib administration. Hence, we speculate that pexidartinib directly induces ATC cell apoptosis, rather than working through immune-related effects as previously reported in FTC [27].

The Warburg effect and elevated oxidative stress are among the characteristics of tumor cells which are usually related with the accumulation of ROS [31]. Cancer cells, including ATC cells, have elevated ROS levels compared to normal cells with compensatory elevated antioxidant mechanisms [32]. Interestingly, excess ROS can also induce cytotoxicity through a series of molecular pathways, in which the ER stress is one of the most typical phenotypes [33]. A widely documented series of small molecule drugs and chemotherapeutic agents induce elevation of ROS and ER stress in cancer cells, including bortezomib, sorafenib, as well as pazopanib, etc. [25,34,35,36]. As there is no known effective target for pexidartinib in ATC cells, we hypothesize that pexidartinib achieves its antitumor effects by mediating excessive oxidative stress and ER stress. Accumulated unfolded proteins in the ER trigger the unfolded protein response (UPR) to increase ER protein folding capacity. ER proteostasis and UPR signaling need to be regulated in a precise and timely manner, in which the aggregation of PDI was identified as an early event and a potential marker during ER stress [37]. Administration of pexidartinib significantly induced the aggregation of PDI, and apoptosis induced by pexidartinib could be alleviated by salubrinal, an agent targeting p-eIF2α [38] which is imperative for ER stress-induced apoptosis. These results indicated that ER stress is a crucial pathway involved in pexidartinib-induced apoptosis in ATC cells. Moreover, significant increases of ROS were observed in ATC cells after administration of pexidartinib and acetylcysteine, a ROS scavenger, which could reverse pexidartinib-induced ER stress and accompanying apoptosis in ATC cells. Thus, ROS is the core mechanism responsible for anti-ATC effects of pexidartinib.

Reactive oxygen species are essential in the natural proliferation of thyroid cells and in the synthesis of triiodothyronine (T3) and thyroxine (T4) in thyroid follicular cells [39]. However, uncontrolled overdose of ROS can lead to oxidative stress, an important element in the etiopathogenesis of a variety of diseases encompassing malignancy and inflammatory diseases [40]. Therefore, thyroid follicular cells require protection against oxidative stress damage through a compensation mechanism. More recent studies have revealed that the anti-oxidative response pipeline centering on Nrf2 is a representative means of defense [41]. Nrf2 has also been reported to be significant in cancers for promoting proliferation [42], and knockdown of the Nrf2 enhances the chemosensitivity in animal tumor models [43]. In an oxidative stress cellular environment, oxidation of specific oxidation-responsive cysteines of Keap1 eliminates its capacity for polyubiquitination and degradation against Nrf2 [44]. As a result, Nrf2 is stabilized and enriched in the nucleus and binds to sequences of antioxidant response elements (AREs) situated in the promoters and enhancers of its respective target antioxidant-related genes [45]. We have observed that after administration with pexidartinib, the nuclear enrichment of Nrf2 in ATC cells was significantly upregulated and interaction of Nrf2 with Keap-1 was weakened, suggesting that Nrf2 is a protective mechanism for pexidartinib-induced oxidative stress and subsequent ER stress and apoptosis induction. Hence, knockdown of Nrf2 in ATC cells significantly enhanced the effects of pexidartinib-induced ATC cells ER stress and apoptosis, indicating that co-administration of pexidartinib with Nrf2 inhibitor might be a better approach for ATC treatment. Hence, the xenograft animal model was set up to evaluate the anticancer characteristics of pexidartinb on ATC as well as the synergistic effects on combination use with ML385, an Nrf2 inhibitor. The results indicated that pexidartinib significantly inhibits ATC cellproliferation in vivo, and co-administration with Nrf2 inhibitor enhances the anti-ATC effects of pexidartinib.

## 5. Conclusions

In conclusion, we found that pexidartinib is a multi-targeted agent with significant anti-tumor activity. Pexidartinib upregulates ROS in ATC cells, thereby inducing ER stress and further causing apoptosis in ATC cells. Furthermore, the protective mechanism of ATC cells triggered the nuclear enrichment of Nrf2, which activated the excitation of antioxidant genes. Therefore, the combination of an Nrf2 inhibitor would enhance the anti-ATC activity of pexidartinib. As a FDA-approved small molecule, pexidartinib has a basic human safety profile, but clinical trials for ATC have not yet been initiated. This study provides ideas and clues for the treatment of ATC with pexidartinib, and relevant clinical trials may further investigate the therapeutic capacity and safety of pexidartinib in human ATC.

## Data Availability

The orininal contributions presented in the study are available under request to correspondence authors.

## Supplementary Materials

The following supporting information can be downloaded at: https://www.mdpi.com/article/10.3390/cancers15010172/s1, Figure S1: Anti-Anaplastic Thyroid Cancer (ATC) Effects and Mechanisms of PLX3397 (pexidartinib), a Multi-targeted Tyrosine Kinase Inhibitor (TKI).
