# Supplementary material for: Anti-Anaplastic Thyroid Cancer (ATC) Effects and Mechanisms of PLX3397 (Pexidartinib), a Multi-Targeted Tyrosine Kinase Inhibitor (TKI)
Source: Cancers (Basel). 2022 Dec 28;15(1):172. doi: 10.3390/cancers15010172 (PMC9817966; doi:10.3390/cancers15010172)
Supplement: Supplementary file 1 [file cancers-15-00172-s001.zip › cancers-2050991-SI.pdf]

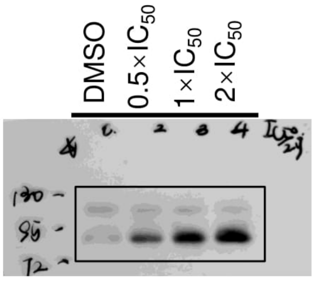

PARP 89 116kDa
CAL-62

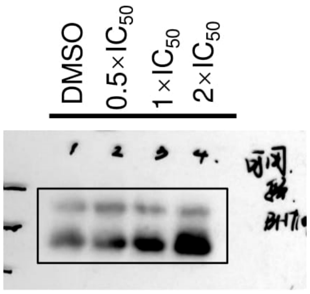

PARP 89 116kDa
BHT101

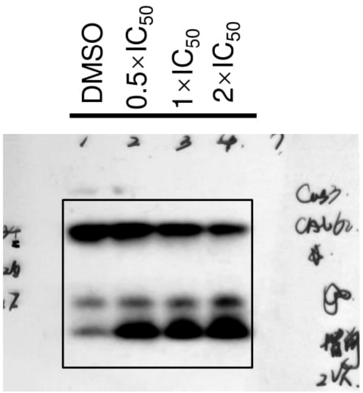

Caspase3 35 17 19kDa
CAL-62

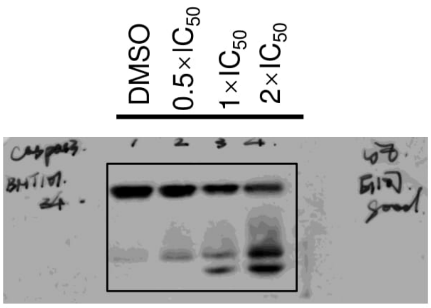

Caspase3 35 17 19kDa
BHT101

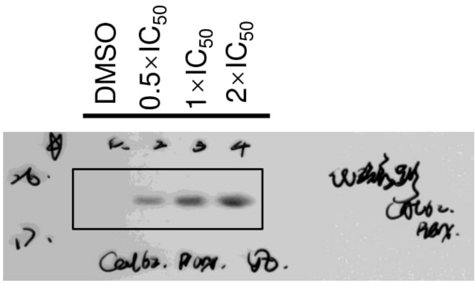

Bax 20kDa
CAL-62

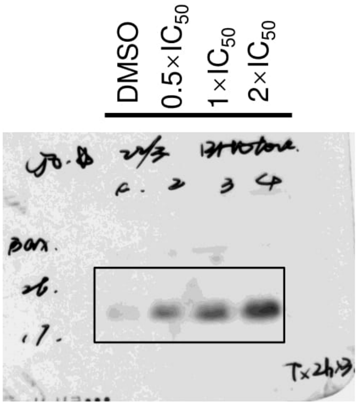

Bax 20kDa BHT101

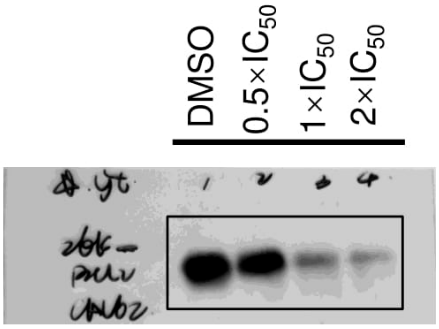

Bcl-2 25kDa
CAL-62

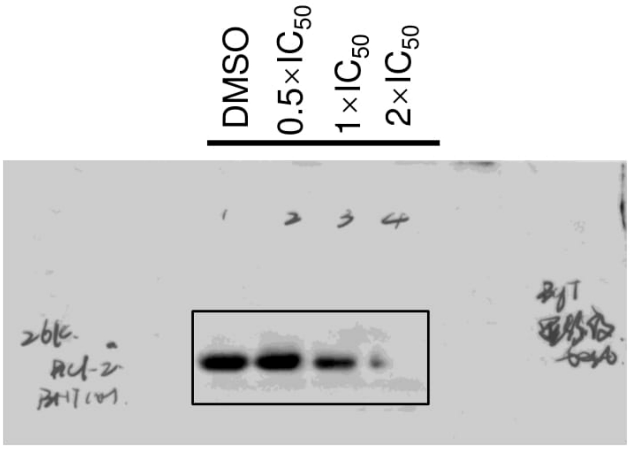

Bcl-2 25kDa
BHT101

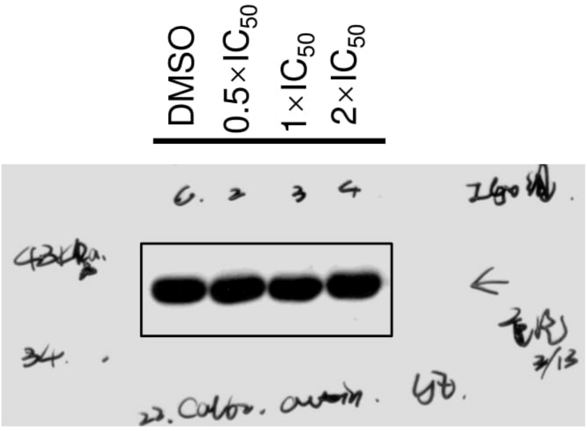

β-actin 42kDa
CAL-62

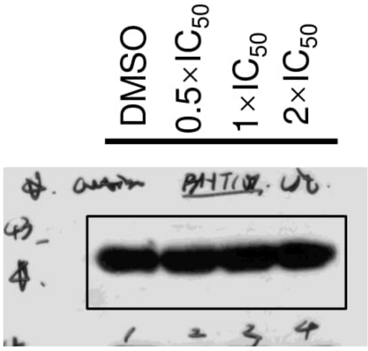

β-actin 42kDa
BHT101

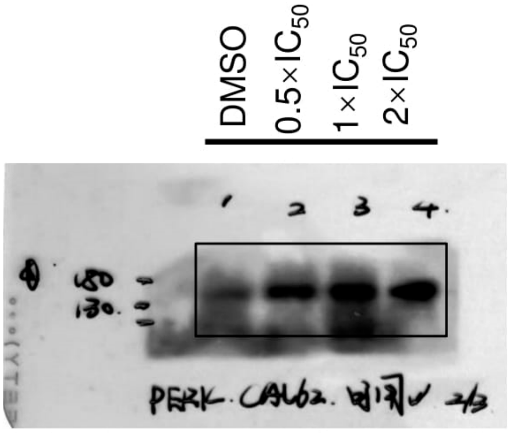

PERK 140
CAL-62

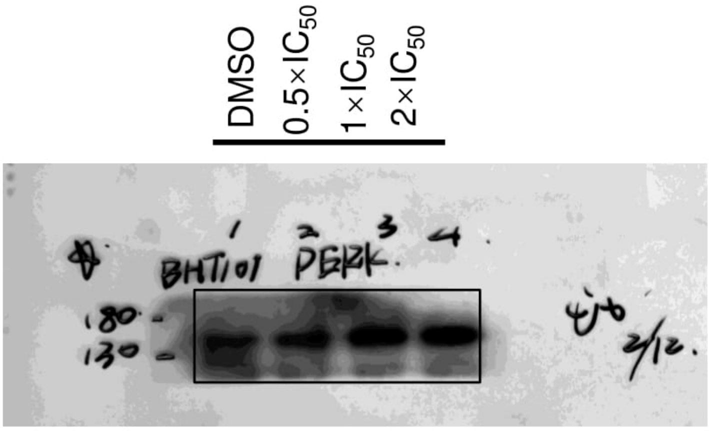

PERK 140
BHT101

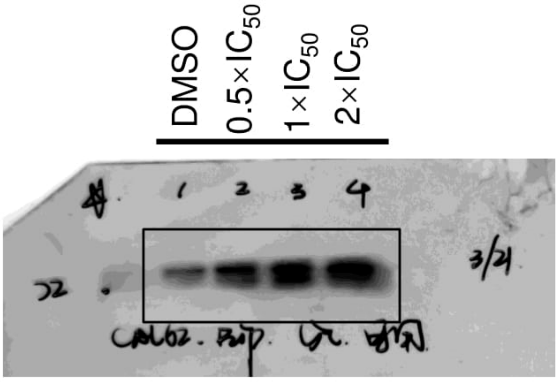

BiP 78

CAL-62

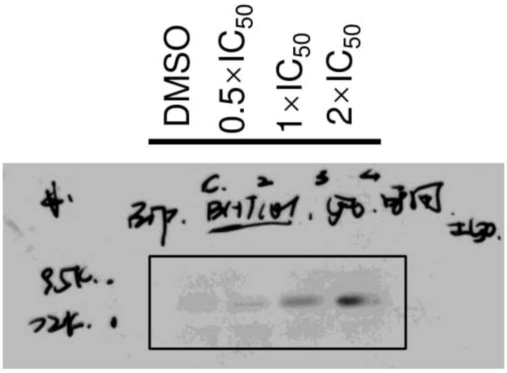

BiP 78

BHT101

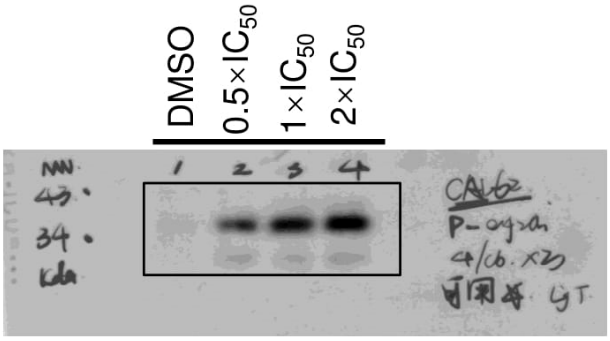

p-eIF2α 36

CAL-62

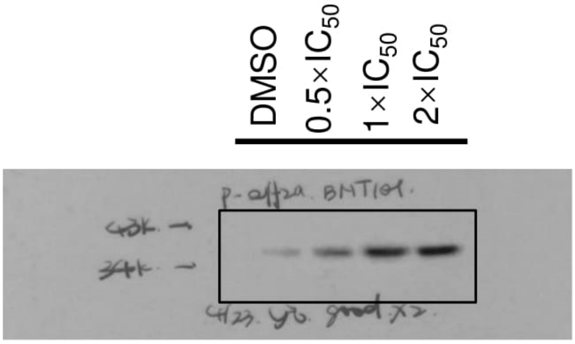

p-eIF2α 36

BHT101

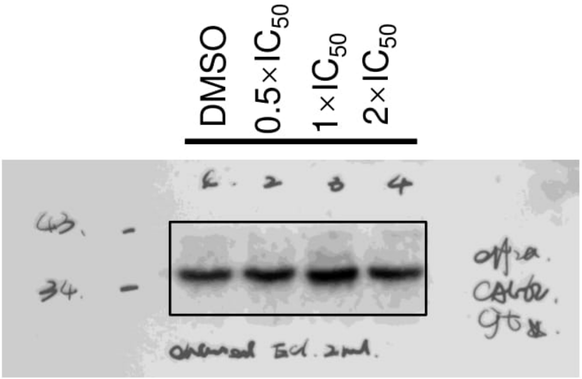

eIF2α 36

CAL-62

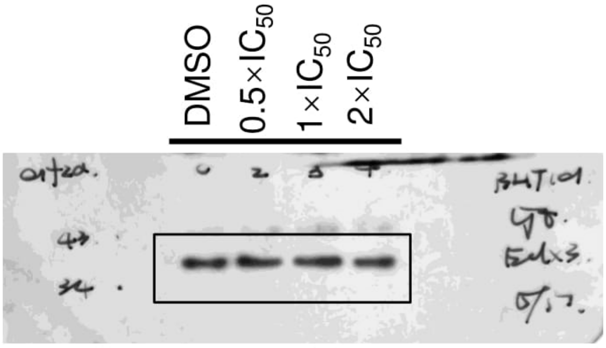

eIF2α 36

BHT101

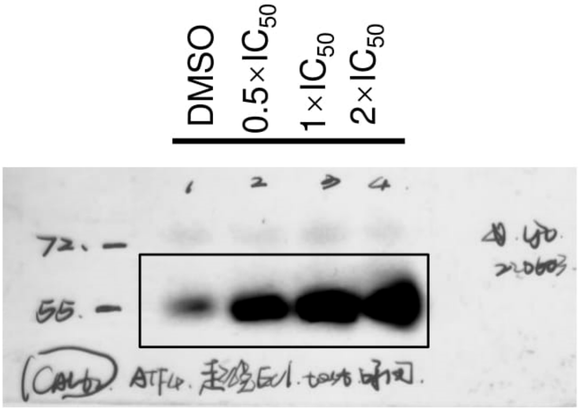

ATF4 55

CAL-62

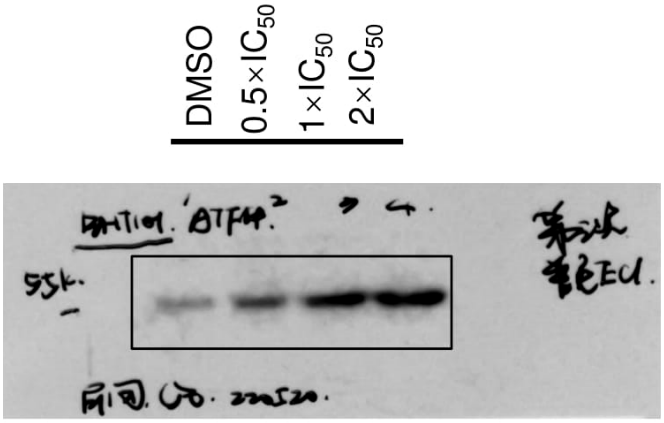

ATF4 55

BHT101

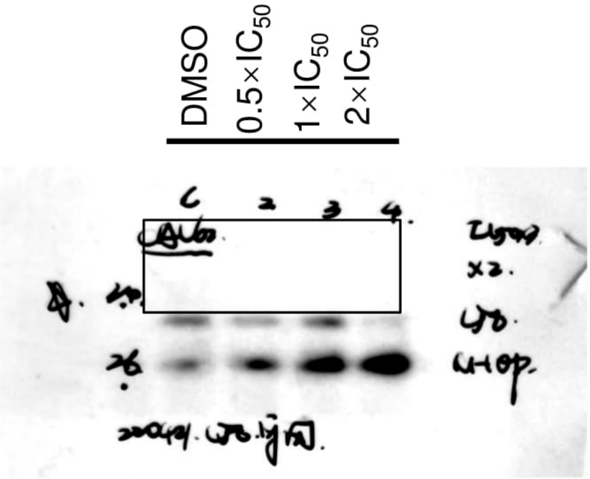

CHOP 29

CAL-62

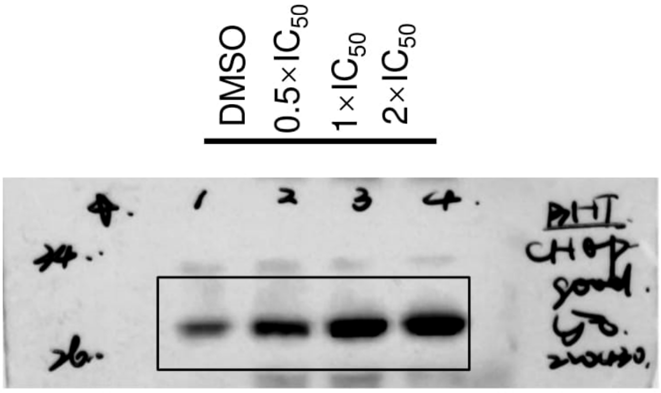

CHOP 29

BHT101

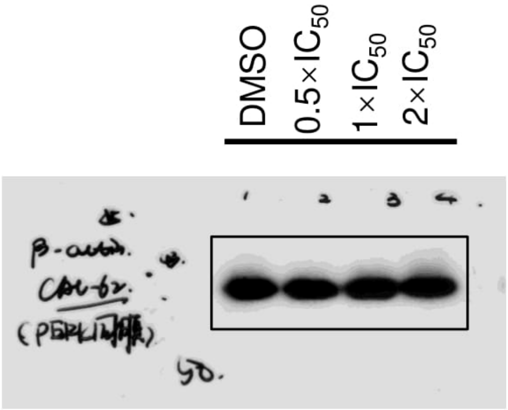

β-actin 42

CAL-62

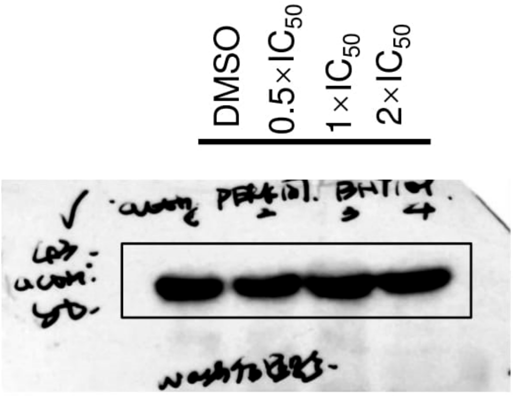

β-actin 42

BHT101

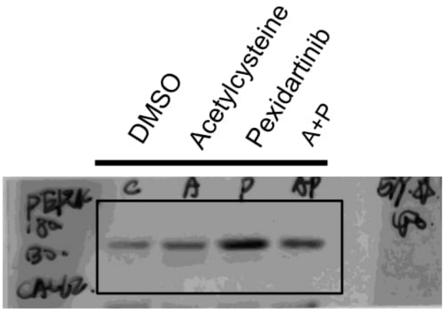

PERK 140

CAL-62

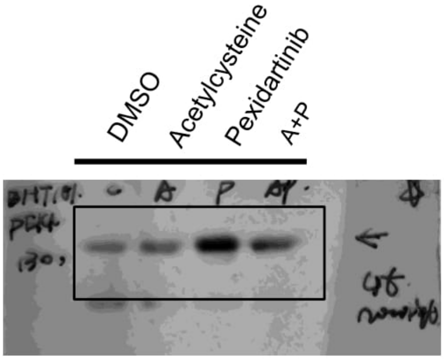

PERK 140

BHT101

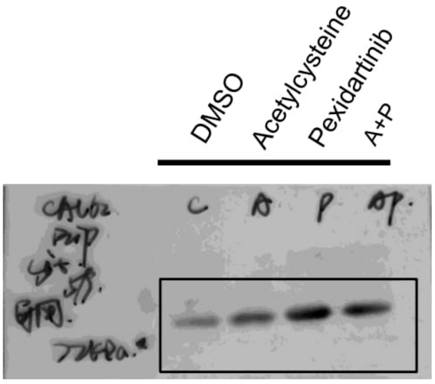

BiP 78

CAL-62

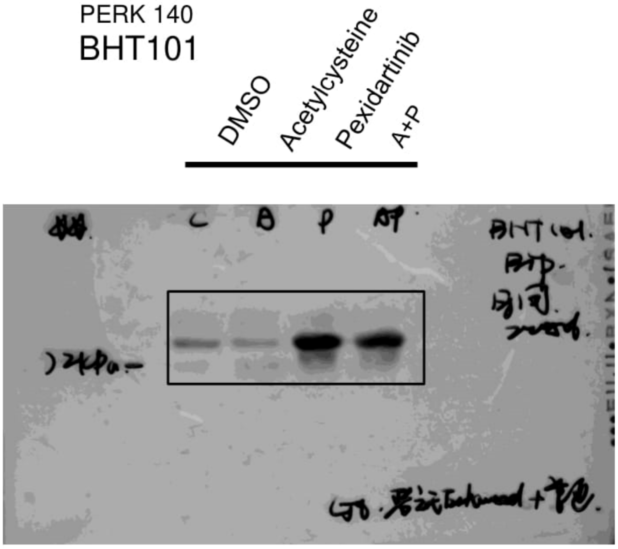

BiP 78

BHT101

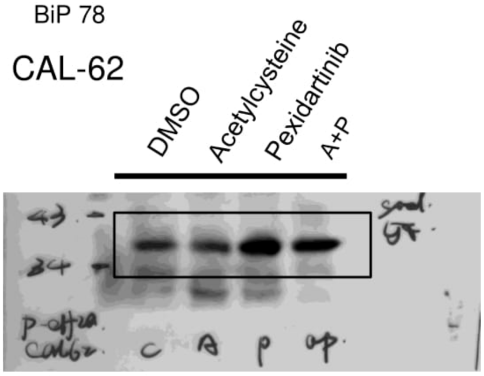

p-eIF2α 36

CAL-62

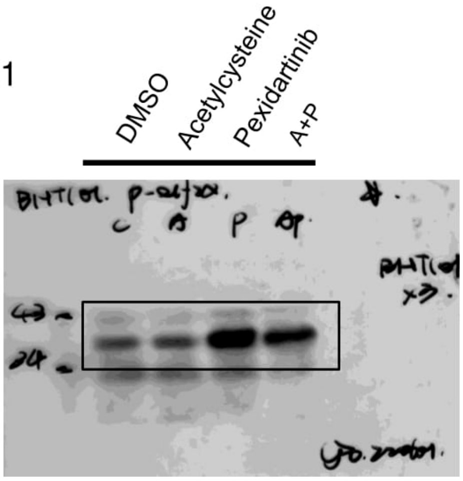

p-eIF2α 36

BHT101

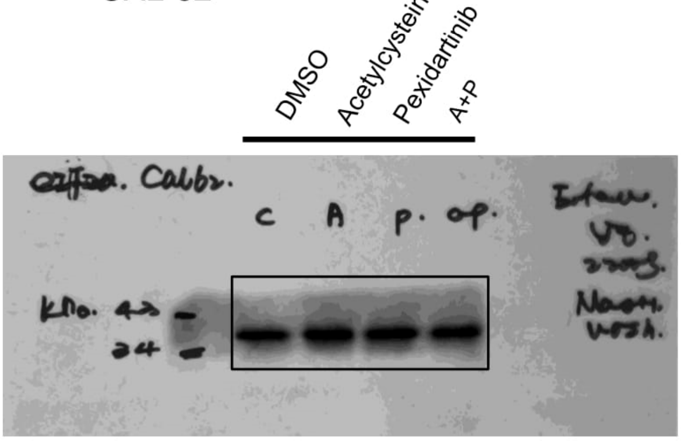

eIF2α 36 CAL-62

BHT101

eIF2α 36

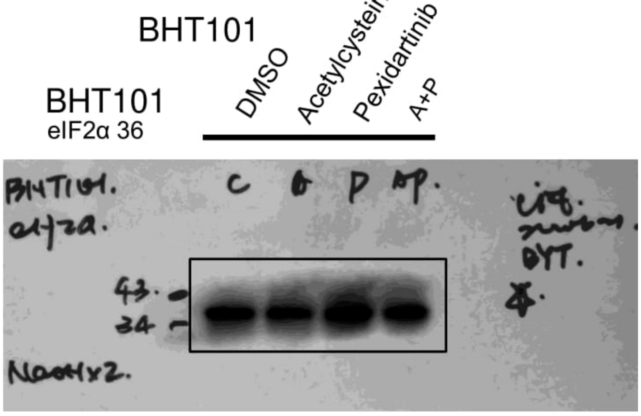

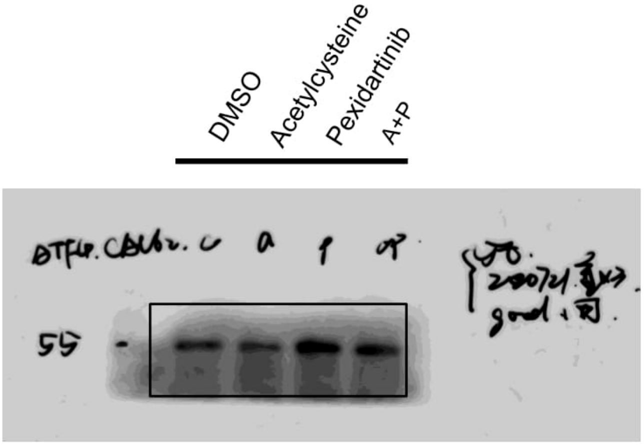

ATF4 55

CAL-62

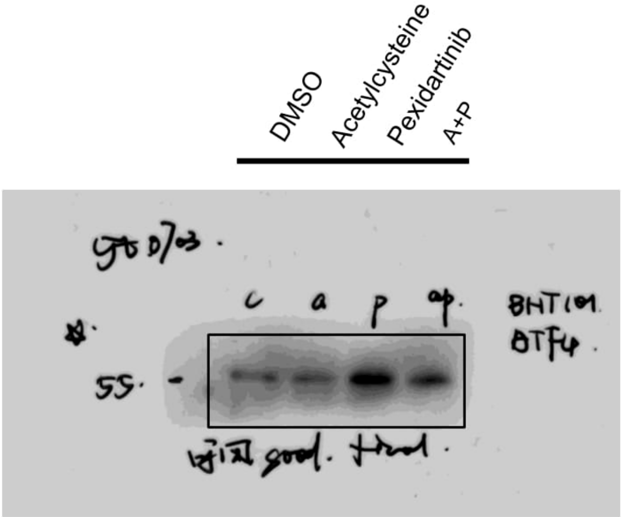

ATF4 55

BHT101

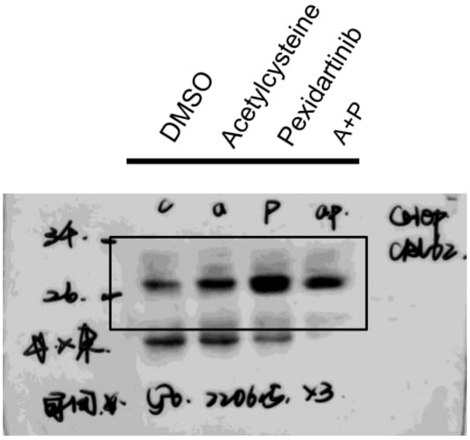

CHOP 29

CAL-62

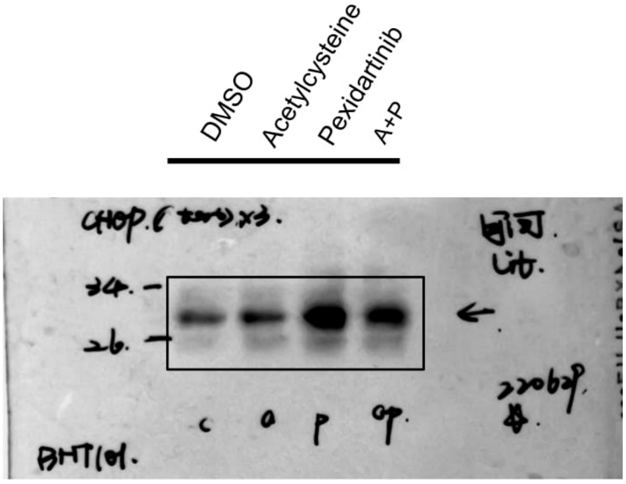

CHOP 29

BHT101

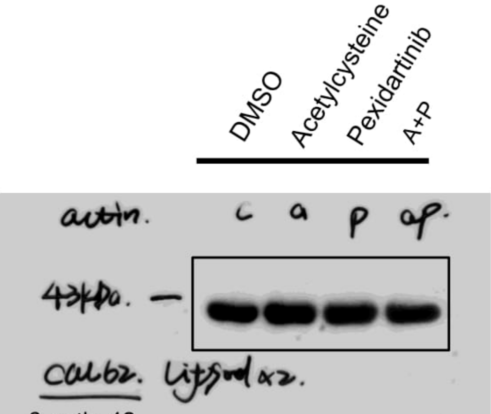

β-actin 42

CAL-62

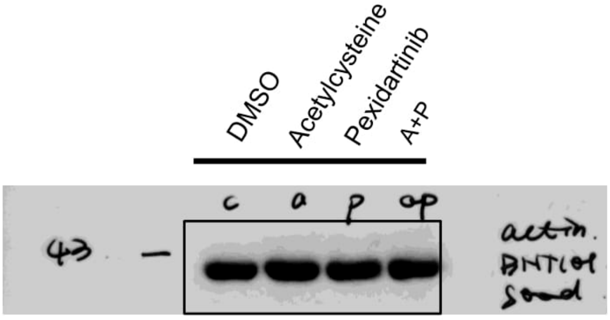

β-actin 42

BHT101

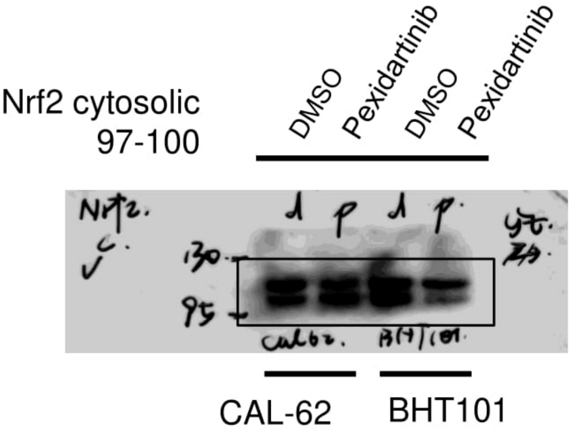
Nrf2 cytosolic
97-100
DMSO
Pexidartinib
DMSO
Pexidartinib
CAL-62
BHT101

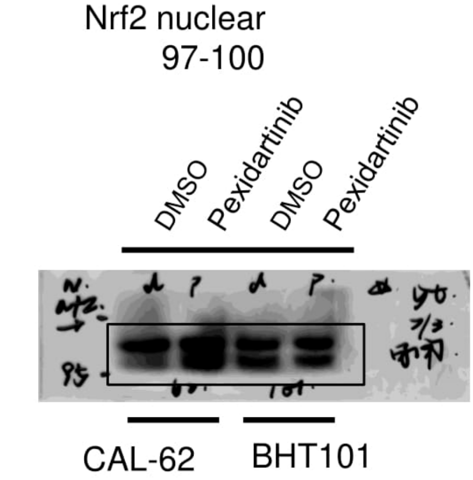
Nrf2 nuclear
97-100
DMSO
Pexidartinib
DMSO
Pexidartinib
CAL-62
BHT101

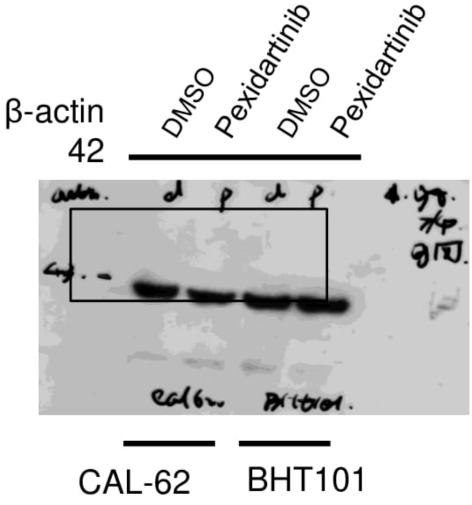
β-actin
42
DMSO
Pexidartinib
DMSO
Pexidartinib
CAL-62
BHT101

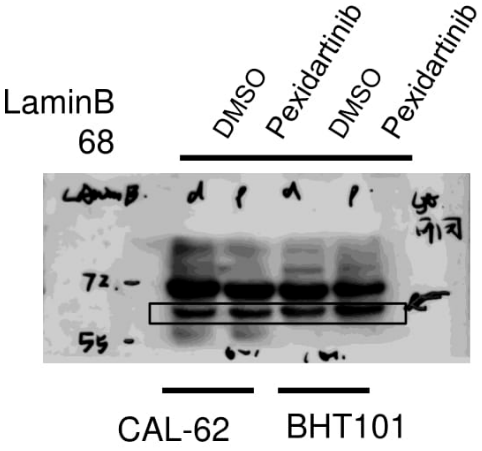
LaminB
68
DMSO
Pexidartinib
DMSO
Pexidartinib
CAL-62
BHT101

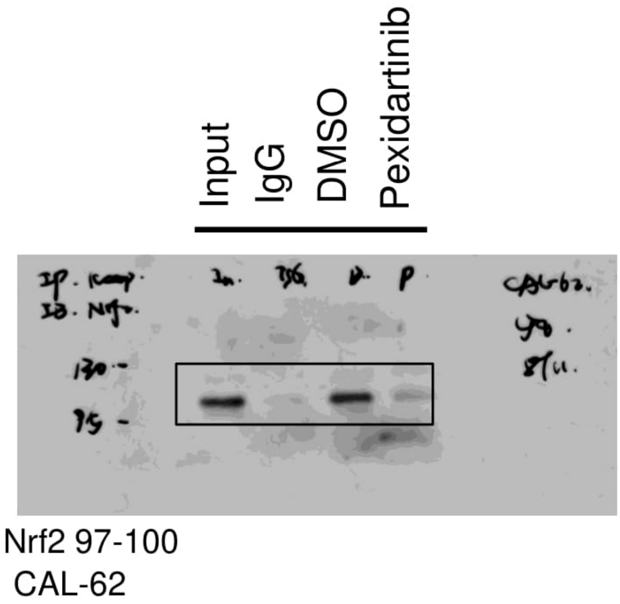
Input
IgG
DMSO
Pexidartinib
Nrf2 97-100
CAL-62

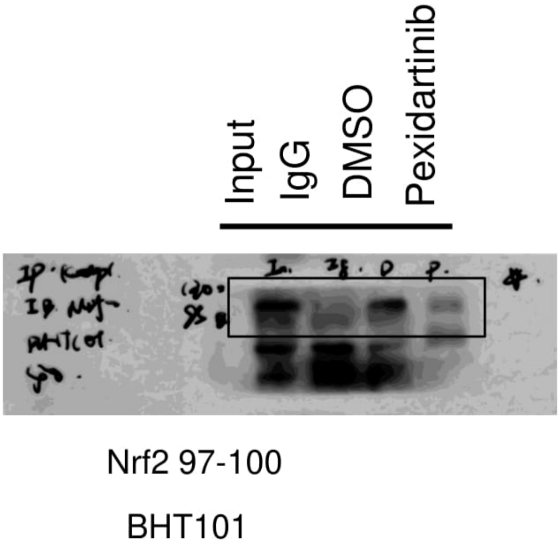
Input
IgG
DMSO
Pexidartinib
Nrf2 97-100
BHT101

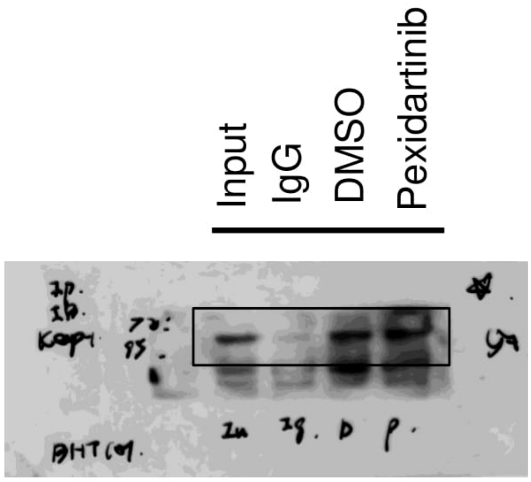

Keap-1 60-64
CAL-62

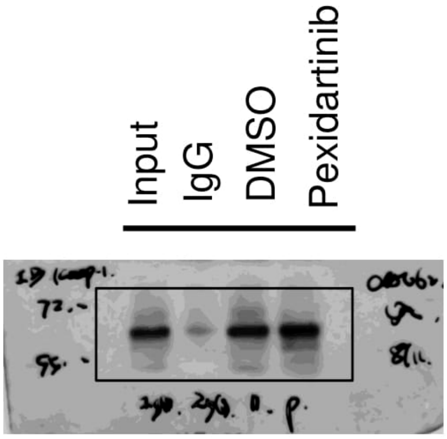

Keap-1 60-64
BHT101

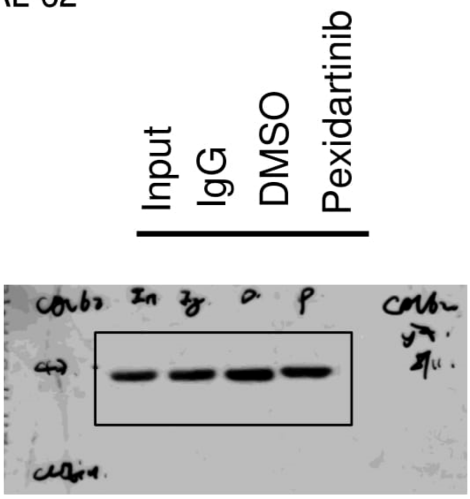

β-actin
42

CAL-62

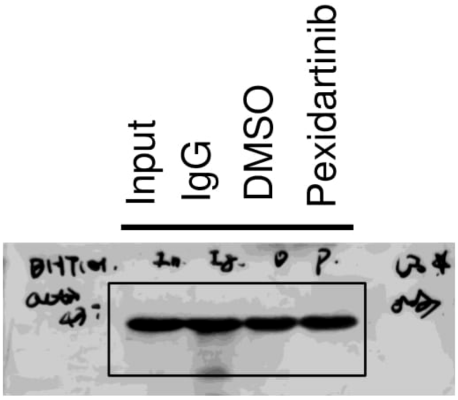

β-actin
42

BHT101

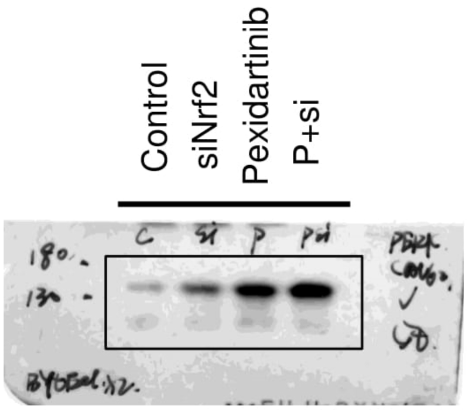

PERK 140 CAL-62

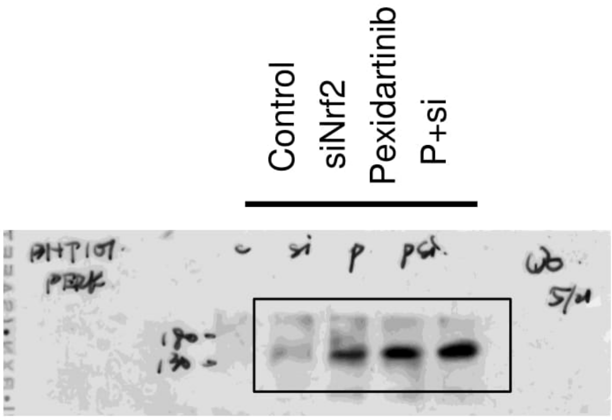

PERK 140 BHT101

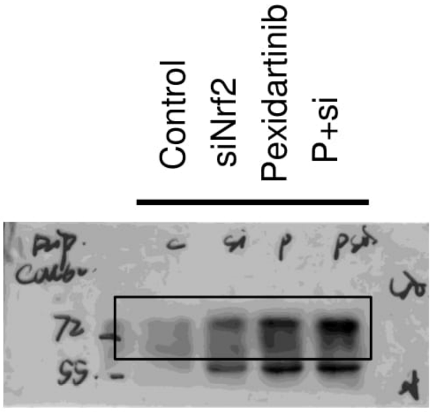

BiP 78

CAL-62

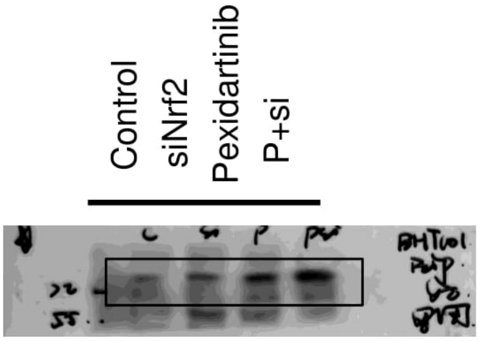

BiP 78

BHT101

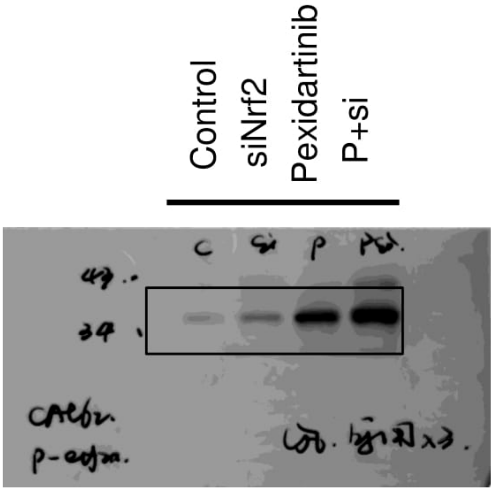

p-eIF2α 36

CAL-62

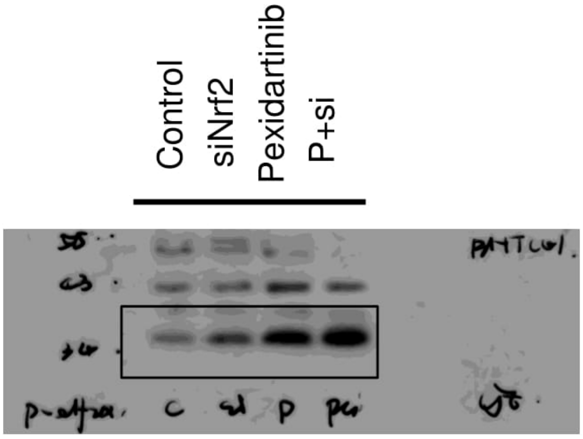

p-eIF2α 36

BHT101

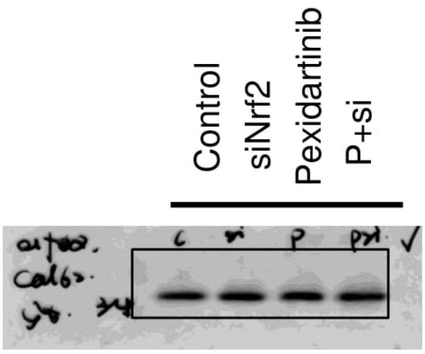

eIF2α 36

CAL-62

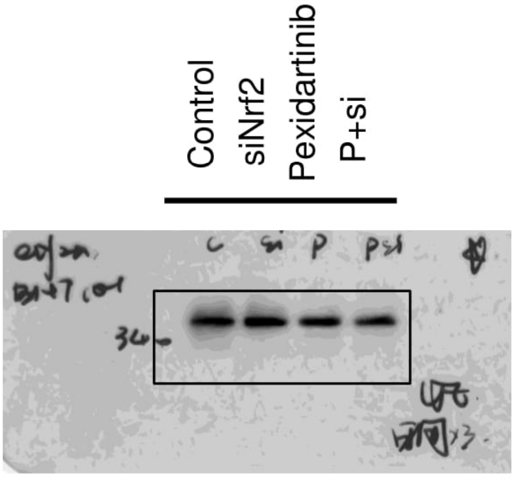

eIF2α 36

BHT101

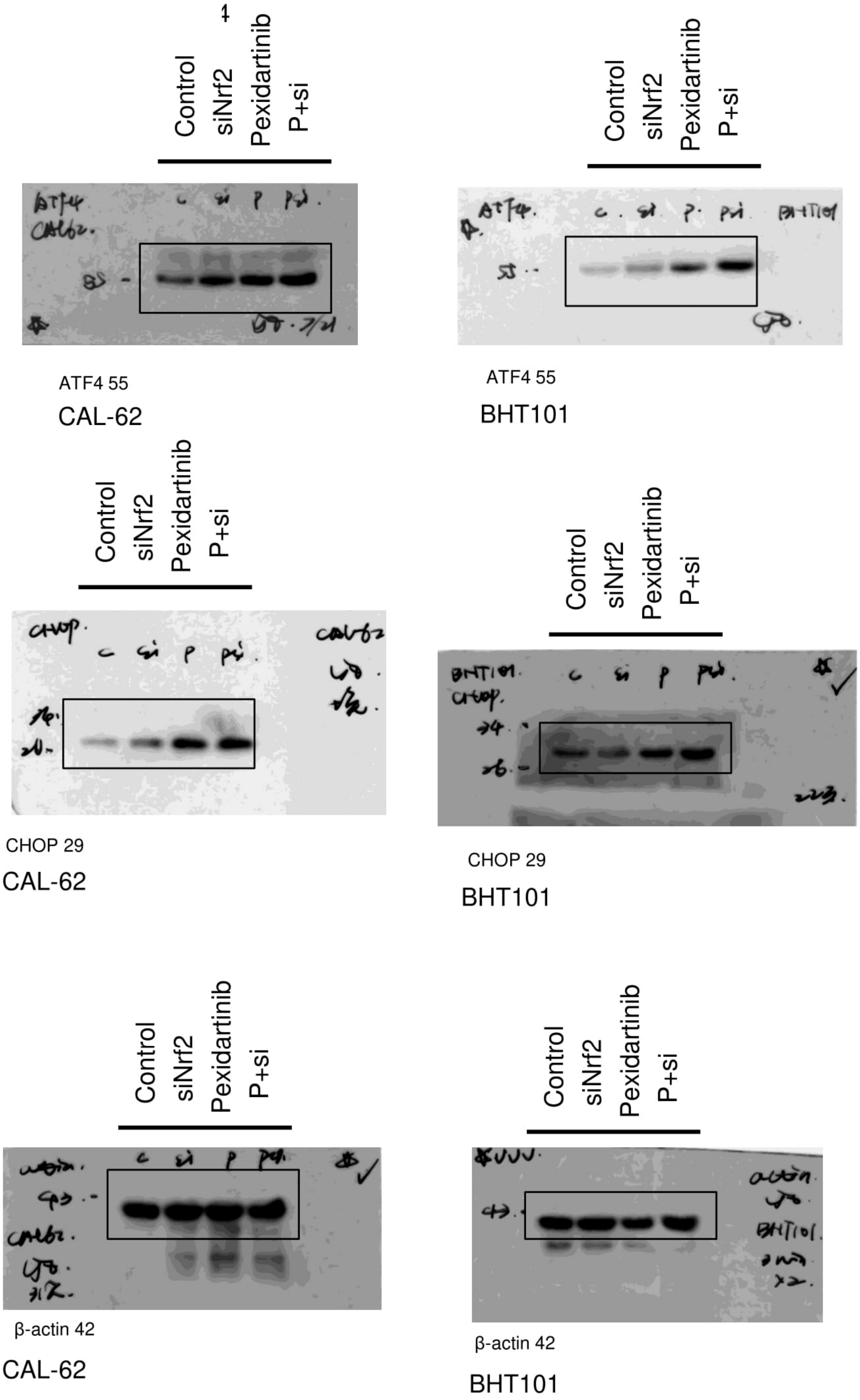

Figure S1. Anti-Anaplastic Thyroid Cancer (ATC) Effects and Mecha-nisms of PLX3397 (pexidartinib), a Multi-targeted Tyrosine Kinase Inhibitor (TKI).
